# Corrosion and wear behaviour of highly porous Ti-TiB-TiN_x_ in situ composites in simulated physiological solution

**DOI:** 10.3906/kim-2001-40

**Published:** 2020-06-01

**Authors:** Fatih TOPTAN

**Affiliations:** 1 CMEMS-UMinho - Center for Microelectromechanical Systems, University of Minho, Guimarães Portugal; 2 Department of Mechanical Engineering, University of Minho, Guimarães Portugal; 3 IBTN/Br -Brazilan Branch of the Institute of Biomaterials, Tribocorrosion and Nanomedicine, Bauru, SP Brasil

**Keywords:** Highly porous Ti, in situ composites, corrosion, wear

## Abstract

Highly porous Ti matrix composites can be a solution for some of the major clinical concerns for the load bearing implants such as low tribocorrosion resistance, stress shielding, and lack of biological anchorage. In order to respond to these needs, highly porous Ti-TiB-TiN_x_ in-situ composites were synthesized by pressureless sintering using BN as reactant and urea as space holder. Corrosion behaviour was investigated at body temperature, in phosphate buffer saline solution (PBS), by measuring open circuit potential (OCP) and cyclic polarization. Wear behaviour was studied in PBS by reciprocating against a 10 mm diameter alumina ball under 3 N of normal load and 1 Hz of frequency. Results showed that the formation of the in-situ reinforcing phases led to an increase on the hardness and on the wear resistance, as well, neither macro porosity nor the reinforcing phases led to localized corrosion.

## 1. Introduction

Metallic biomaterials are still indispensable since at least approximately 70% of biomedical implants are produced from them [1–3]. Among the available metallic biomaterials, Ti and its alloys are the most popular ones due the combination of excellent electrochemical properties and good mechanical behaviour, however, their poor wear resistance is a major concern [3]. It is known that poor wear resistance of Ti and its alloys results in the excessive release of wear debris and metallic ions in physiological conditions that can create adverse local and systemic effects [4–6]. Many studies have been performed in order to improve the wear behaviour of Ti and its alloys, including coatings, surface modifications, and incorporation of hard reinforcement phases [7,8].

Apart from low wear resistance, stress shielding, arising from the Young’s modulus mismatch between bone and the implant material, and lack of biological anchorage for bone ingrowth are other concerns for biomedical implants [9,10]. In these respects, highly porous metals can be beneficial on reducing the Young’s modulus down to the values found in the range of bone, enabling bone in-growth providing a mechanical interlocking, thus, a better fixation [11–14]. Besides, highly porous structures also provide other advantages such as promoting transportation of nutrients and vascularization, as well as offering lower density and weight [11,15].

Due to the high melting point of Ti and its high reactivity, powder-based routes were widely employed to produce highly porous Ti. Within several powder-based processing routes, powder metallurgy (P/M) with space holder technique has been widely studied owing to its low-cost, ease of fabrication, and versatility to adjust pore size and level of porosity [16,17]. Nevertheless, additive manufacturing techniques have been used in recent years on processing highly porous or cellular structures offering further advantages on the control of geometry and pore architecture [11].

While offering aforementioned advantages, in addition to the low wear resistance, highly porous Ti has some disadvantages, mainly as reduced mechanical strength especially on higher pore levels [18]. Besides, porosity can affect the electrochemical behaviour in a way to occur localized corrosion due to the exhaustion of oxygen in the small, close pores [19], or leading to formation of a heterogenous passive film on the most inner pore surfaces [20]. In order to overcome these issues, some studies are available in the literature investigated the techniques such as surface modification [21] and incorporation of hard phases to produce metal matrix composites (MMCs) [22–25]. Within MMCs, in situ composites offer several advantages for highly porous Ti, such as formation of clean, continuous matrix/reinforcement interfaces with stronger bonding, ease to deal with smaller reinforcement phases with more homogenous distribution, and lower processing costs [26]. However, limited number of studies are available in the literature regarding in situ highly porous Ti. Within those, Blackwood et al. [25] produced highly porous Ti-based composites having porosity levels varying between approximately 14%–27% by using Ti and graphite powders, utilizing different pressures on P/M, however, reported increased corrosion rates on the composite samples. Liu et al. [22] produced highly porous Ti-TiC composites having 50% of porosity by P/M with space holder technique and reported improved compressive strength on the highly porous composites. Gain et al. [23] produced Ti-ZrO_2_ nanocomposites having 50% of nominal porosity, by P/M with space holder technique, and reported reduced grain size and increased hardness for the composites. Tang et al. [24] produced Ti6Al4V matrix, in situ formed TiC reinforced composites having approximately 25% of porosity by microwave sintering using multiwalled carbon nanotubes as reactant (also acted as a microwave susceptor) and reported increased hardness and compressive strength for the composites.

The author and his coworkers had previously synthesized dense Ti-TiB-TiN_x_ in situ hybrid composites by reactive hot pressing and reported significantly improved dry sliding wear [27] and tribocorrosion [28] behaviour for the composites. In the present work, Ti-TiB-TiN_x_ in situ hybrid composites were produced with 30% nominal porosity (i.e. space holder volume fraction) by P/M with space holder technique, and their corrosion and wear behaviour were studied in a physiological solution.

## 2. Materials and methods

Highly porous Ti-TiB-TiN_x_ in situ composites having 30% of nominal porosity were processed by P/M with space holder technique utilizing Ti (Grade 2, Alfa Aesar, Thermo Fisher (Kandel) GmbH, Kandel, Germany) and BN (Sigma-Aldrich Chemie GmbH, Taufkirchen, Germany) particles (23:1 Ti:BN weight ratio) having 26.7 and 1 μm average particle sizes, respectively, together with urea particles (Scharlab, S.L., Barcelona, Spain) sieved under 500 μm, as space holder. The powder blend mixed by ball milling at 130 rpm during 4 h under argon atmosphere. Green compacts were obtained by uniaxially pressing at 350 MPa for 2 min in a nitrided stainless steel die having zinc stearate lubricated inner surfaces. Afterwards, space holder was removed in a horizontal tubular furnace by keeping the green compacts under Ar atmosphere at 450 °C during 3 h, according to the differential thermal analysis and thermal gravimetric analysis (DTA/TG) that had been presented elsewhere [20]. Then, sintering was performed in a horizontal tubular furnace under high vacuum (<10^-5^ mbar) at 1100 °C for 3 h with 5 °C/min heating and cooling rates. Before characterization and testing, cylindrical samples having 12 mm of diameter, 5 mm of thickness were grinded using diamond grinding disks and final polishing was done with colloidal silica suspension (0.04 μm). Finally, samples were placed upside down in a beaker using a clip and ultrasonically cleaned during 15 min in propanol and 10 min in distilled water to avoid accumulation of contaminants into the macro pores.

Electrochemical tests consisting of measurement of OCP and cyclic polarization were employed in a three-electrode electrochemical cell adapted from American Society for Testing and Materials (ASTM) G3-89 standard, containing 150 mL of PBS with the composition of 0.2 g/L KCl, 0.24 g/L KH_2_PO_4_, 8 g/L NaCl, and 1.44 g/L Na_2_HPO_4_. The cell was positioned in a climate chamber keeping the temperature constant at body temperature (37 °C), as well, functioning as a Faraday cage. The reference electrode (saturated Ag/AgCl), the counter electrode (Pt net), and the working electrode (samples having a geometric exposed area of 0.38 cm^2^) were connected to Gamry Reference 600+ Potentiostat/Galvanostat/Zero resistance ammeter (ZRA). Cyclic polarization was employed after 24 h of stabilization in PBS at body temperature, by performing a polarization scan starting from 100 mV below OCP with 0.5 mV/s scanning rate till 1000 mV where the sweep direction was reversed. All potentials were presented with respect to Ag/AgCl.

Wear tests were performed against a 10 mm diameter alumina ball, in PBS solution, using a tribometer (PLINT TE 67/R) in reciprocating mode, under 3 N normal load (corresponding to 0.59 GPa maximum Hertzian contact pressure for dense grade 2 Ti), 1 Hz of frequency, 4 mm of total stroke length, and 30 min of sliding time. After testing, samples were cleaned ultrasonically following the procedure described above, and kept in adesiccator for further characterization.

Phase analysis of the as-processed highly porous samples was performed by X-ray diffraction (XRD, Bruker D8 Discover diffractometer, Bruker Corporation, Billerica, MA, USA) with a Cu Kα radiation source. As-processed and tested samples were characterized using an optical microscope (OM, Leica DM2500, Leica Microsystems GmbH, Wetzlar, Germany) and field emission gun scanning electron microscope (FEG-SEM, FEI Nova 200, FEI Company, Hillsboro, Oregon, USA) equipped with energy dispersive X-ray spectroscopy (EDS, EDAX-Pegasus, EDAX Inc., Mahwah, NJ, USA). One group of composite samples were deeply-etched by Kroll’s reagent (HF:HNO_3_:H_2_O) in order to observe the in situ reinforcing phases on FEG-SEM. Pore size distribution and pore percentages were measured on the OM images by image analysis with Image J 1.37v software. Vickers microhardness values were measured by a microhardness tester (DuraScan, Emco-Test Prüfmaschinen GmbH, Kuchl, Austria) under 0.05 kgf during 15 s over five indentations per sample. After wear tests, thickness of the wear tracks was measured as an average of three measurements on each wear track.

All tests and measurements were repeated at least 3 times for ensuring repeatability and the respective results were presented as average ±standard deviation.

## 3. Results and discussion

**Figure 1:**
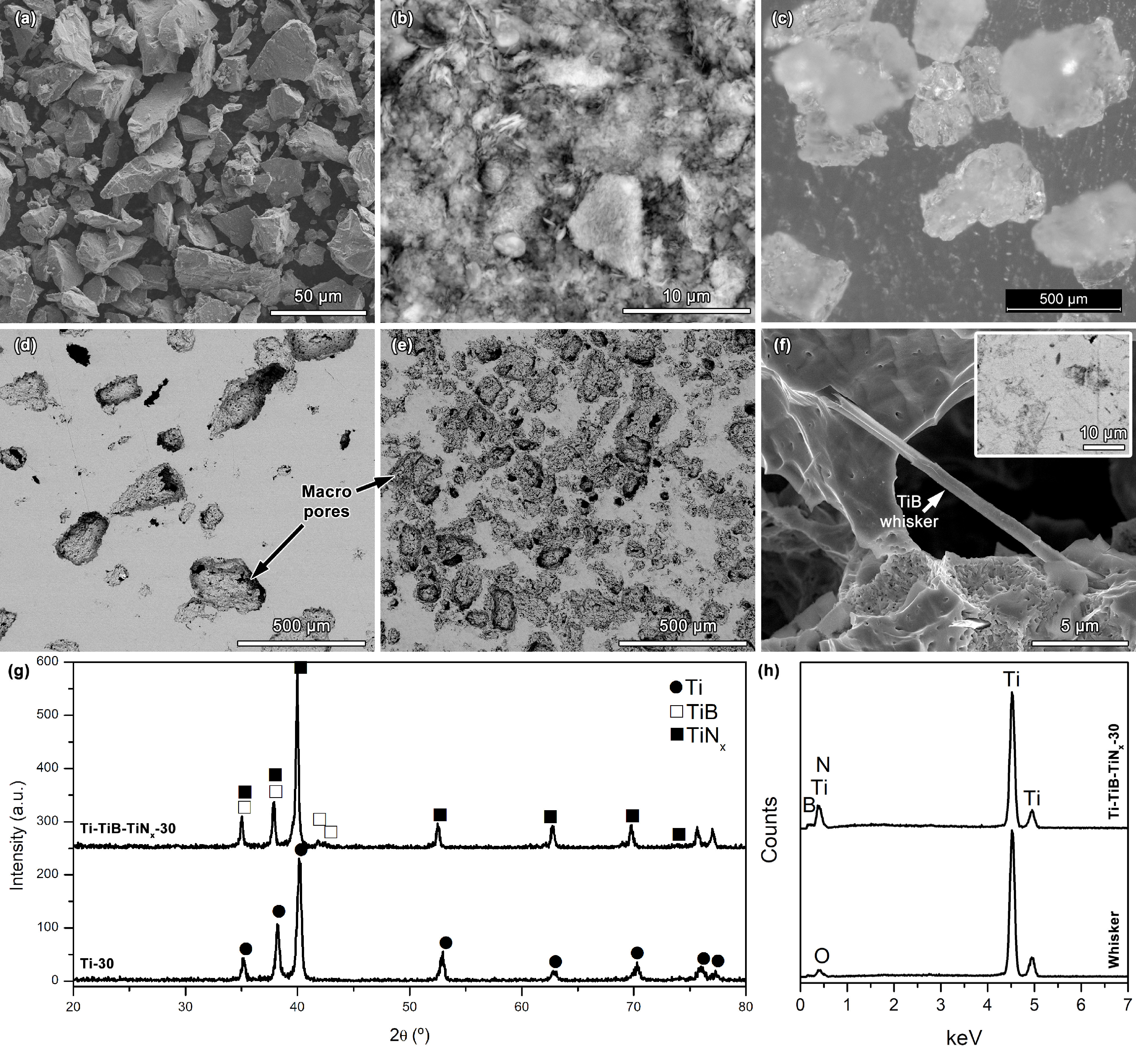
Characterization before and after processing: Backscattered electron (BSE) SEM images of a) Ti particles and b) BN particles (agglomerates), c) OM image of urea particles; BSE images of the as-processed d) Ti-30 and e) Ti-TiB-TiN_x_-30 samples, f) higher magnification secondary electron (SE) SEM image obtained on the deeply-etched composite sample showing a TiB whisker, together with the BSE image of the polished composite outmost surface (insert) g) XRD spectra of the as-processed samples, and h) EDS spectra taken on the polished composite (insert on f) and TiB whisker (f).

Starting from angular shaped Ti, BN, and urea powders (Figures 1a–1c), highly porous Ti and Ti-TiB-TiN_x_ in situ composites having 30% of nominal porosity (hereafter, it will be referred as Ti-30 and Ti-TiB-TiN_x_ -30, respectively) were produced (Figures 1d–1f). Detailed microstructural analysis of the dense Ti-TiB-TiN_x_ in situ composite synthesised by using the same Ti:BN weight ratio at the same temperature had previously been reported elsewhere [27] where Ti, TiB whiskers, and substoichiometric TiN_x_ phases where identified on the structure based on SEM/EDS and XRD analyses, and the formation mechanism of each in situ reinforcing phase were discussed. Ti (card number of 00-044-1294), TiB (card number of 01-073-2148) and TiN_x_ (card numbers of 01-082-7366 and 01-082-7367) phases were identified on XRD (Figure 1g) for the samples of the present work. While TiB whiskers were clearly visible on the deeply-etched composite samples (Figures 1f and 1h), although it was detected by XRD (Figure 1g), and N was detected on EDS (Figure 1h), it was not easy to observe the TiN_x_ phase by SEM, that needs further examinations by transmission electron microscope (TEM). Due to the strengthening effect given by the in situ reinforcing phases, hardness was increased on the highly porous composites (650 ±183 HV_0.05_) as compared with the unreinforced highly porous samples (506 ±95 HV_0.05_). The author and his coworkers had previously processed dense Ti-TiB-TiN_x_ composites by reactive hot pressing, using the same Ti:BN weight ratio, and reported 316 ±27 and 736 ±65 HV_30_ hardness values for the unreinforced Ti and the composite, respectively [27]. Higher hardness values found on the Ti-30 samples can be related with the possible contamination by the residual urea [29,30]. On the other hand, relatively lower values on the composites may be related with the elevated residual porosity.

Porosity of Ti-30 and Ti-TiB-TiN_x_-30 samples were measured as 23 ±5% and 53 ±3%, respectively. Unreinforced samples exhibited lower values as compared to the nominal value, that can be related with the shrinkage that also had been reported in the literature for highly porous materials processed by P/M with space holder technique [20,31,32]. On the other hand, Laptev et al. [31] reported that during compaction, space holder particles form bridges between Ti particles, deteriorating the densification of the Ti framework, that can also contribute the differences between the measured and the nominal porosity. Since composite green compacts also contain BN reactant particles, this bridging effect can be expected to increase. Besides, the presence of reactant particles, and furthermore, the in situ reactions may alter the sintering kinetics and sintering mechanisms. Therefore, combination of all those effects may explain the elevated porosity percentage obtained on the composite samples. Nevertheless, it is worthy to note that all measurements were done on the surfaces that were subjected to corrosion and wear, that is the upper surface of the sample, where the porosity values might differ from the bulk porosity values due to inhomogeneities through the bulk of the samples. Thus, future studies should contain optimization of the highly porous composites by dilatometric and calorimetric studies together with high temperature XRD analysis, and the homogeneity of the porosity through all sample volume should be defined by microscopic computed tomography (micro-CT) examinations.

**Figure 2:**
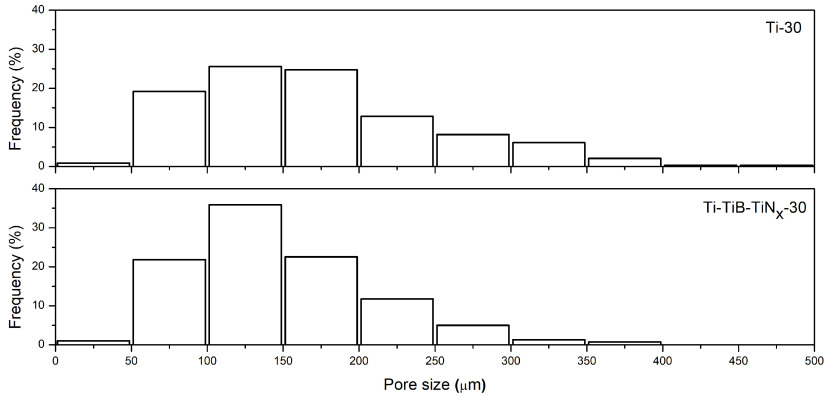
Pore size distribution.

Figure 2 presents the pore size distribution for each group of samples where it can be seen that 25.5% and 35.9% of pores were found in the range of 100–150 μm for the unreinforced and composite samples, respectively. On the other hand, average pore size was defined as 171 ±79 μm and 149 ±60 μm for the unreinforced and composite samples, respectively. The function of the macro pores is to provide available sites for osteoblast attachment and proliferation, to enable transport of body ?uids, and finally to achieve micromechanical interlocking through the ingrowth bone, ensuring the stability of the implant [33,34]. It is known from the literature that different size of pores is beneficial for different purposes, such as bone ingrowth, mechanical strength, and Young’s modulus [35]. Although there is no consensus in the literature regarding the optimum pore size, the range of 100-500 μm is suggested by several authors as to be beneficial for bone ingrowth [11,33]. Therefore, it can be stated that 80.6% and 77.3% of the pores in the Ti-30 and Ti-TiB-TiN_x_ -30 samples, respectively, were in the range that had been reported to be favourable for bone ingrowth.

**Figure 3:**
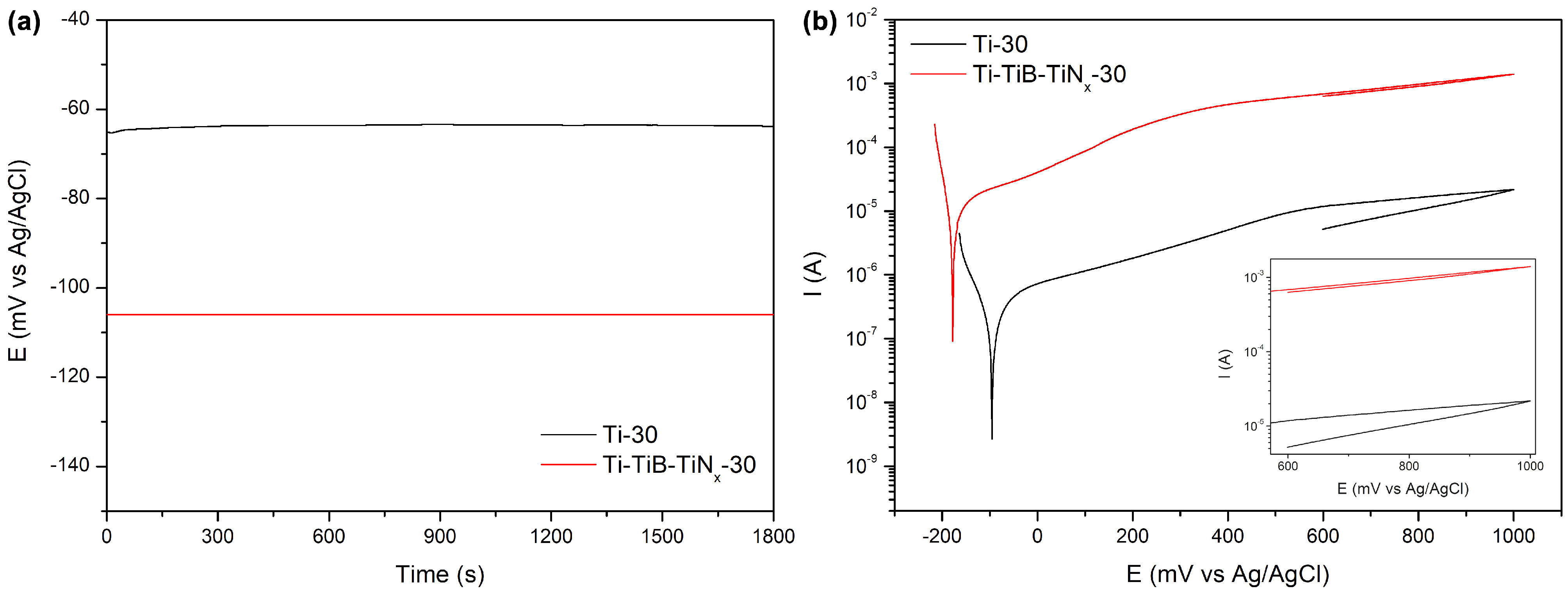
Representative evolution of OCP during the last 30 min of immersion (a) and cyclic polarization curves (b).

One of the main concerns on the behaviour of the highly porous metallic materials as a biomaterial is their corrosion behaviour. It is known that porosity can alter the electrochemical response of highly porous Ti being strongly influenced by the fraction, type (i.e. open or close porosity), and geometry of pores. Eventually, localized corrosion can be seen on small, isolated pores due to consumption of oxygen, or passivation plateau may be lost due to heterogeneities formed on the passive film on the macro pore surfaces [20,34,36]. Representative graphs showing the OCP evolution during the last 30 min of immersion and cyclic polarization curves are given in Figures 3a and 3b, respectively, and average values of the last 30 min of OCP, corrosion potential (E_corr_), and passivation current (I_pass_) taken at 600 mV are given in table. Results showed lower corrosion potential and higher I_pass_ values for the composites, however, cyclic polarization curves indicated that porosity did not lead to a localized corrosion on Ti since lower current values observed during reverse scan, compared to the respective values recorded on the forward scan. Nevertheless, very slowly increased anodic current values were observed on the passive region that was probably related to the heterogeneities on the passive film formed on the pores, as also previously shown by the author and his coworkers [20] by electrochemical impedance spectroscopy (EIS) measurements on highly porous Ti having different levels of porosity including 30% of nominal porosity as studied in the present work.

**Table:** Average values of the last 30 min of OCP, E_corr_, and I_pass_.

Sample	OCP (mV)	E_corr_ (mV)	I_pass_ (μA)
Ti-30	−65 ± 4	−95 ± 6	8.6 ±3. 6
Ti-TiB-TiN_x_-30	−99 ± 21	−148 ± 46	840.0 ±317.9

It is known that incorporation or formation of reinforcing phases can affect the electrochemical response of the matrix metal through galvanic coupling between the matrix metal and the reinforcement phase, increased electrochemical activity at the matrix/reinforcement interface, or degradation of the interphases [8]. Since composite samples presented similar shape of curves to the ones obtained on the unreinforced samples, it may be stated that incorporation of the in situ reinforcement phases did not directly affect the corrosion mechanism significantly, but it seems that indirectly affected the electrochemical response by resulting in higher porosity percentage, that obviously induced a significant increase on the exposed area to the electrolyte, that eventually led to higher current values. It is worthy to stress that instead of current density, current values were presented on Figure 3b and table, since it was hard to determine precisely the metallic exposed area to the electrolyte. Considering the differences on porosity level between the unreinforced and reinforced samples, it is obvious that the real exposed area on the composite can be significantly higher than the unreinforced samples. Thereby, further studies should determine the real surface area by using micro-CT, and the electrochemical results should be interpreted by normalizing with the real exposed area that will give a clearer insight on the corrosion kinetics where, possibly, the difference between the unreinforced and reinforced highly porous Ti will be minimized.

Studies in the literature showed that porosity can play either beneficial or detrimental role on the wear behaviour of highly porous metals. Beneficial effects occur due to ejection of wear debris into the pores, reducing the occurrence of third-body abrasive wear [37–40] or by decreased coefficient of friction (COF) due to the increased lubrication, since pores act as reservoir for lubricants such as body fluids in case of biomedical implant applications [38,41]. However, presence of pores decreases the real contact area between the highly porous metal and the counterpart resulting in increased contact pressures on the outmost surfaces that can increase the severity of wear [37,38,40,42,43].

**Figure 4:**
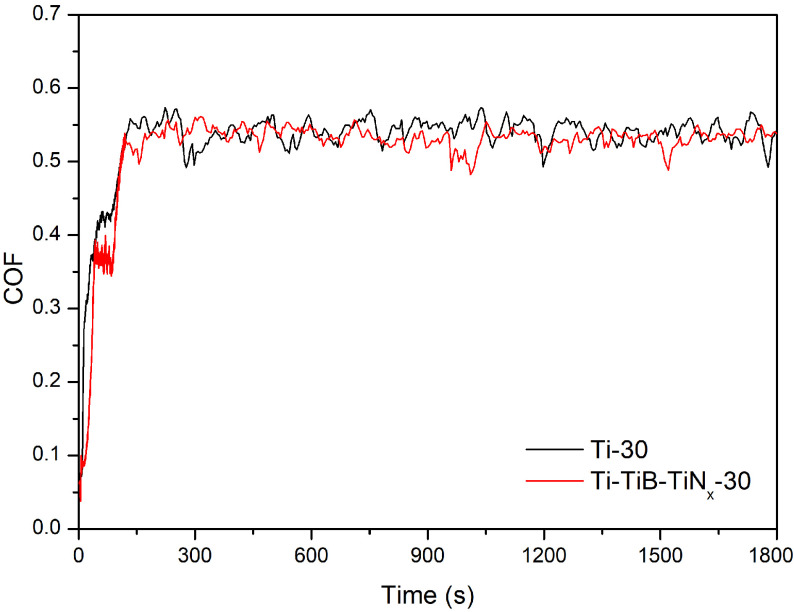
Representative evolution of COF.

Figure 4 presents representative evolution of COF during sliding period. Interestingly, although the samples had different characteristics such as pore size/fraction and hardness, both unreinforced and reinforced highly porous samples exhibited similar evolution of COF values where after a short run-in period, the values reached to a steady state. The average COF values of the three tests taken at the last 1500 s of sliding were 0.52 ±0.04 and 0.54 ±0.06 for the unreinforced and reinforced samples, respectively, showing very similar values for the steady state. These results suggested that the competition between topography and hardness may be the reason of obtaining similar COF values, and to clarify the contribution of each factor, further studies should be performed on samples having different levels of porosity fraction, pore size, and reinforcement volume fraction. However, although unreinforced and reinforced samples exhibited similar COF, even though local contact pressures on the contact zones of the composite increased due to its higher pore fraction as compared to the unreinforced samples, wear resistance was significantly improved on the composite samples that can be seen on the average wear track thickness values measured as 0.54 ±0.02 mm and 0.36 ±0.04 mm for the unreinforced and reinforced samples, respectively.

**Figure 5:**
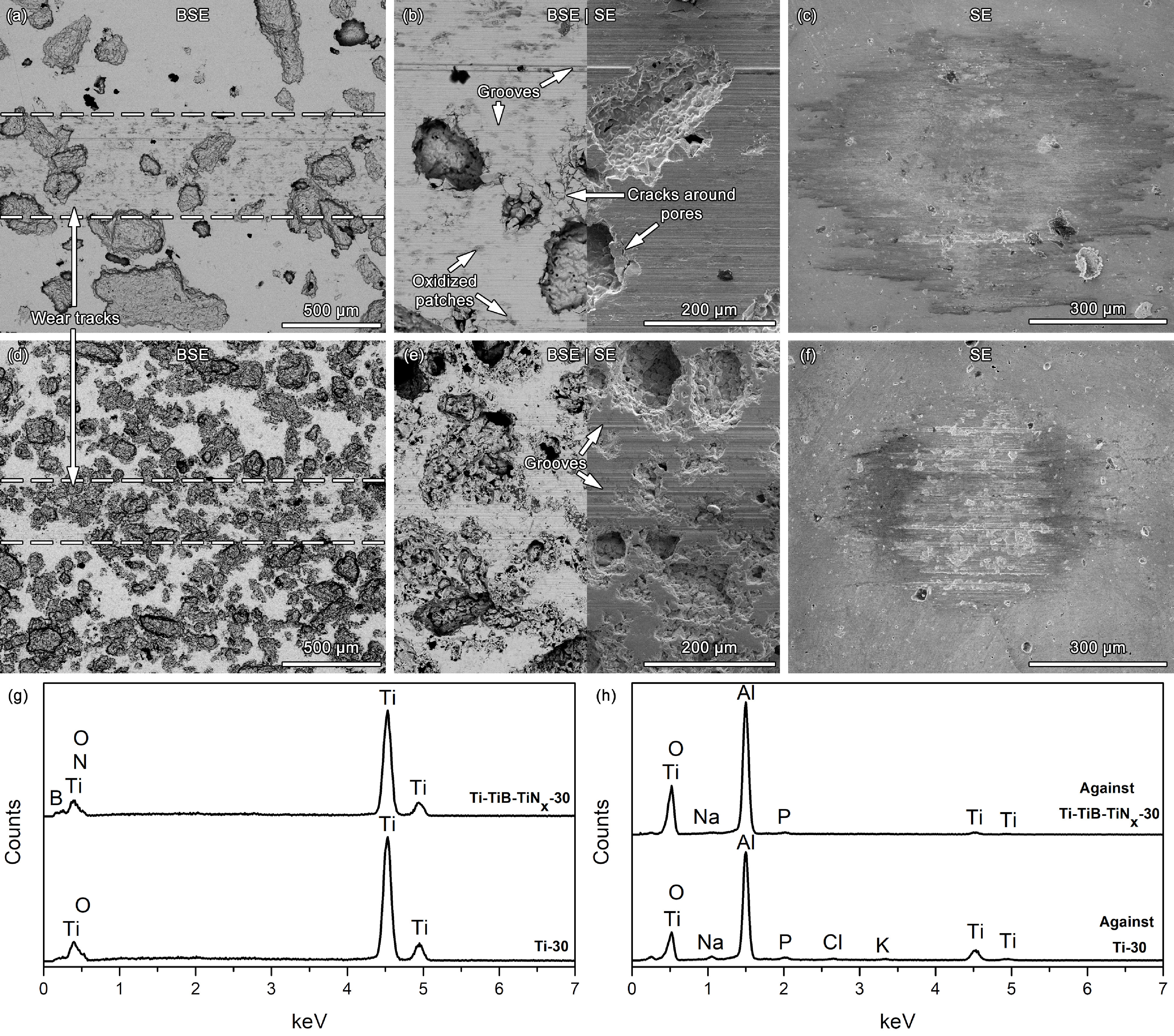
Characterization of the worn surfaces: Lower (a, d) and higher (b, e) magnification SEM images of the worn surfaces and respective counter-material surfaces (c, f) for Ti-30 and Ti-TiB-TiN_x_-30 samples, respectively, together with EDS analyses taken from the worn sample (g) and counter-material (h) surfaces.

Figure 5 shows the representative SEM images and the EDS analyses of the worn sample and countermaterial surfaces. Both unreinforced and reinforced samples presented sliding grooves as parallel to the sliding direction, whereas EDS analysis revealed transfer of Ti to the counter-material, being the indication of abrasive and adhesive wear, respectively, which are the typical wear mechanisms reported for Ti and its alloys [28,44]. When the worn Ti-30 surfaces were compared with the reported [28] dense Ti surfaces worn under 9 g/L NaCl solution, against the same counter-material, under both lower and higher normal loads, it was clearly noticed that the oxidized patches, that are being formed after repetitive transfer of material between the mating surfaces, were sparse on the Ti-30 surfaces, possibly due to ejection of most of the loose oxidized debris into the macro pores, as had also been reported by the author and his co-workers for the highly porous Ti [21]. The same was also observed for the Ti-TiB-TiN_x_ -30 samples, where, although oxygen was detected on both reinforced and unreinforced worn surfaces, the composite worn surfaces were almost free of a visible tribolayer or oxidized patches that may be mainly linked with the increased amount of porosity on the composite samples. On the other hand, SEM images and EDS analysis of the worn counter-material surfaces revealed less transferred Ti on the composite samples indicating decreased influence of adhesive wear on the composite. Besides, on Ti-30 samples, cracks were observed around the macropores which were not evident on the composite samples that can indicate the increased strength given by the in situ reinforcing phases. Thus, based on these facts, it can be stated that the dominant wear mechanism was the combination of abrasive and adhesive wear for both groups of samples, however, the influence of adhesive wear was significantly reduced on the composite samples.

Although this study showed some promising results on development of wear-resistant highly porous in situ composites by using a low-cost processing technique, it has some limitations and requires further studies to fully understand the properties of highly porous Ti-TiB-TiN_x_ in situ composites for load bearing biomedical implant applications. First of all, as also stated above, the real metallic exposed area should be defined and should be taken in consideration on interpreting the electrochemical response. Second, the effect of pore size/fraction and reinforcement volume fraction to friction and wear should be understood by testing different levels of each parameter. Moreover, long term immersion tests should be performed by periodic EIS measurements in order to monitor the quality of the passive film through a prolonged time of immersion. Furthermore, tribocorrosion behaviour of these highly porous composites should be explored in order to understand the degradation mechanisms under the simultaneous action of corrosion and wear. On the other hand, mechanical behaviour, particularly compression, shear, and bending strength should be defined together with Young’s modulus. Finally, biological response of these composites should be studied and, in the end, by combining all electrochemical, tribo-electrochemical, mechanical, and biological data, optimum composition including the size and fraction of porosity and reinforcement should be defined and the processing conditions should be optimized.

## 4. Conclusions

Highly porous Ti-TiB-TiN_x_ in-situ hybrid composites were produced by presureless sintering using BN as reactant and urea as space holder. It can be concluded within the testing conditions that the formation of the in situ reinforcing phases led to an increase on the hardness, as well as on the wear resistance in PBS. Electrochemical studies in PBS revealed no localized corrosion neither due to macro porosity nor due to the formation of the in situ phases. Therefore, highly porous Ti-TiB-TiN_x_ in situ composites can be promising for load-bearing implants and worth further investigation to explore its application in vivo.
